# Short-Term Peri-Procedural Dapagliflozin for Prevention of Contrast-Induced Acute Kidney Injury in Diabetic Patients Undergoing Coronary Computed Tomographic Angiography: A Prospective Randomized Double-Blind Placebo-Controlled Study

**DOI:** 10.1159/crm/abtag003

**Published:** 2026-08-20

**Authors:** Tian Zheng, Lifeng Zhang, Jianxin Hu, Xiaoshu Cheng, Yuxuan Zhong, Xiao Huang, Lianggeng Gong, Biming Zhan

**Affiliations:** a Department of Radiology, The Second Affiliated Hospital, Jiangxi Medicine College, Nanchang University, Nanchang, China; b Department of Cardiovascular Medicine, Jiangxi Medicine College, The Second Affiliated Hospital, Nanchang University, Nanchang, China

**Keywords:** Sodium-glucose cotransporter 2 inhibitors, Dapagliflozin, Contrast-induced acute kidney injury, Coronary computed tomographic angiography, Diabetes mellitus, Renal oxygenation

## Abstract

**Introduction:**

Sodium-glucose cotransporter 2 (SGLT2) inhibitors have established long-term cardiorenal benefits in patients with type 2 diabetes mellitus. However, their short-term peri-procedural role in preventing contrast-induced acute kidney injury (CI-AKI) after coronary computed tomographic angiography (CCTA) remains uncertain, and their acute effects on renal oxygenation may be complex.

**Methods:**

This prospective, randomized, double-blind, placebo-controlled single-center study screened 352 diabetic patients scheduled for CCTA. A total of 335 patients were randomized, and 322 patients with complete baseline and post-CCTA creatinine assessment were included in the modified intention-to-treat analysis. Participants received dapagliflozin 10 mg once daily or matched placebo beginning 3 days before CCTA and continuing through the 72-h post-CCTA follow-up. CI-AKI was defined according to prespecified serum creatinine-based criteria within 72 h after contrast exposure. Serial renal indices and exploratory biomarker variables were summarized at available time points, and logistic regression was restricted to a parsimonious covariate set because of the limited number of events.

**Results:**

The modified intention-to-treat analysis included 322 patients, with 161 patients assigned to each group. CI-AKI occurred in 29 patients overall (9.0%), with a significantly lower incidence in the dapagliflozin group than in the placebo group (6/161 [3.7%] vs. 23/161 [14.3%]; odds ratio, 0.23; 95% confidence interval, 0.09–0.59; *p* = 0.001 by Fisher’s exact test). Baseline renal indices, including serum creatinine, estimated glomerular filtration rate, blood urea nitrogen, neutrophil gelatinase-associated lipocalin, and baseline eGFR category distribution, were comparable between groups. At 72 h after CCTA, the dapagliflozin group had lower serum creatinine and higher eGFR than the placebo group. KDIGO creatinine-based AKI stage distribution did not differ significantly between groups (*p* = 0.064), and no patient required new dialysis or kidney replacement therapy during the 72-h follow-up. Exploratory secondary biomarker analyses showed lower blood urea nitrogen and neutrophil gelatinase-associated lipocalin levels at 72 h in the dapagliflozin group.

**Conclusion:**

In diabetic patients undergoing CCTA, short-term peri-procedural dapagliflozin was associated with a lower incidence of creatinine-defined CI-AKI and improved early post-contrast renal function profiles. By focusing on a noninvasive CCTA population, this study expands the potential application of SGLT2 inhibition from invasive coronary procedures to intravenous contrast-enhanced cardiac CT, suggesting a novel adjunctive approach for renal risk reduction in selected diabetic patients. Larger multicenter trials are needed to validate these findings and define the patients most likely to benefit.

**Highlights**

- Short-term peri-procedural dapagliflozin was associated with a lower incidence of creatinine-defined CI-AKI in diabetic patients undergoing CCTA.
- The analysis cohort was transparently defined; baseline renal indices and eGFR categories were comparable between treatment groups, and baseline LVEF was reported as median (interquartile range) to avoid distortion by low-end values.
- Consistent Scr-eGFR trajectories, study-defined CI-AKI, KDIGO creatinine-based AKI severity, and exploratory BUN/NGAL profiles were reported to contextualize early renal changes after CCTA.
- The findings should be interpreted as preliminary and hypothesis-generating rather than as evidence supporting routine peri-procedural SGLT2 inhibitor use.

## Introduction

Coronary computed tomographic angiography (CCTA) is an established noninvasive imaging modality for the evaluation of coronary artery disease, plaque characterization, and cardiovascular risk stratification [1, 2]. Despite advances in scanner technology and contrast protocols, iodinated contrast administration remains integral to CCTA and may be associated with acute deterioration in renal function in susceptible patients [3, 4].

Diabetes mellitus is an important susceptibility factor for post-contrast acute kidney injury because it is commonly accompanied by endothelial dysfunction, renal microvascular disease, oxidative stress, chronic inflammation, altered intrarenal hemodynamics, and impaired renal oxygen handling [5, 6]. Although the absolute renal risk after intravenous contrast-enhanced CT is generally lower than that after invasive coronary procedures, the risk is not uniform across patient groups and may increase substantially in patients with reduced baseline estimated glomerular filtration rate (eGFR) or coexisting diabetic kidney disease [7, 8].

Risk stratification studies in coronary procedures have also underscored the importance of baseline renal and clinical vulnerability in contrast-associated AKI [9, 10]. Sodium-glucose cotransporter 2 (SGLT2) inhibitors were developed as glucose-lowering agents but have since shown broad cardiorenal benefits, including slower chronic kidney disease progression and fewer heart failure events [11, 12]. Emerging observational and interventional studies suggest that SGLT2 inhibition may reduce AKI risk in selected cardiovascular settings, including invasive coronary procedures [13, 14]. However, whether short-term peri-procedural SGLT2 inhibition can reduce contrast-induced acute kidney injury (CI-AKI) after CCTA has not been established.

The biological rationale is also not straightforward. SGLT2 inhibition may improve proximal tubular energetics and modulate inflammatory and oxidative pathways, but it may also shift sodium delivery toward distal nephron segments, potentially increasing medullary transport workload under some acute conditions [15]. Therefore, peri-contrast SGLT2 inhibitor use requires direct clinical evaluation rather than extrapolation from chronic cardiorenal outcome trials. This study evaluated whether short-term peri-procedural dapagliflozin reduces creatinine-defined CI-AKI in diabetic patients undergoing CCTA and explored clinical factors associated with CI-AKI risk.

## Materials and Methods

### Study Design and Ethical Approval

This was a prospective, randomized, double-blind, placebo-controlled single-center study conducted at the Second Affiliated Hospital of Nanchang University. The study was designed and reported in accordance with the Consolidated Standards of Reporting Trials (CONSORT) 2025 statement, and the completed CONSORT checklist is provided as online supplementary material (for all online suppl. material, see https://doi.org/10.1159/crm/abtag003). The trial was registered in the Chinese Clinical Trial Registry (ChiCTR2500098100; https://www.chictr.org.cn/) on March 3, 2025, before enrollment of the first participant on May 5, 2025. The implementation and recruitment dates initially entered in the registry reflected the anticipated study schedule. Study initiation was delayed because completion of registry review and operational preparations, including study medication and matched placebo preparation, randomization procedures, and staff coordination, took longer than originally anticipated. No participant provided trial-specific informed consent, was randomized, or received the study intervention before registration.

### Study Population and Analysis Population

Adult patients with type 2 diabetes mellitus scheduled for CCTA were prospectively screened during the actual recruitment period from May 5, 2025, to April 6, 2026. A total of 352 patients were screened, 335 were randomized, and 322 patients with complete baseline and post-CCTA serum creatinine (Scr) measurements were included in the modified intention-to-treat analysis. Post-randomization exclusions were attributable to incomplete essential data or failure to complete the 72-h post-CCTA renal follow-up, as summarized in Figure 1. The modified intention-to-treat population included randomized participants who received the assigned study medication and had post-procedural creatinine data available for primary endpoint assessment. Participants without outcome ascertainment could not contribute to the primary endpoint analysis.

**Fig. 1.**
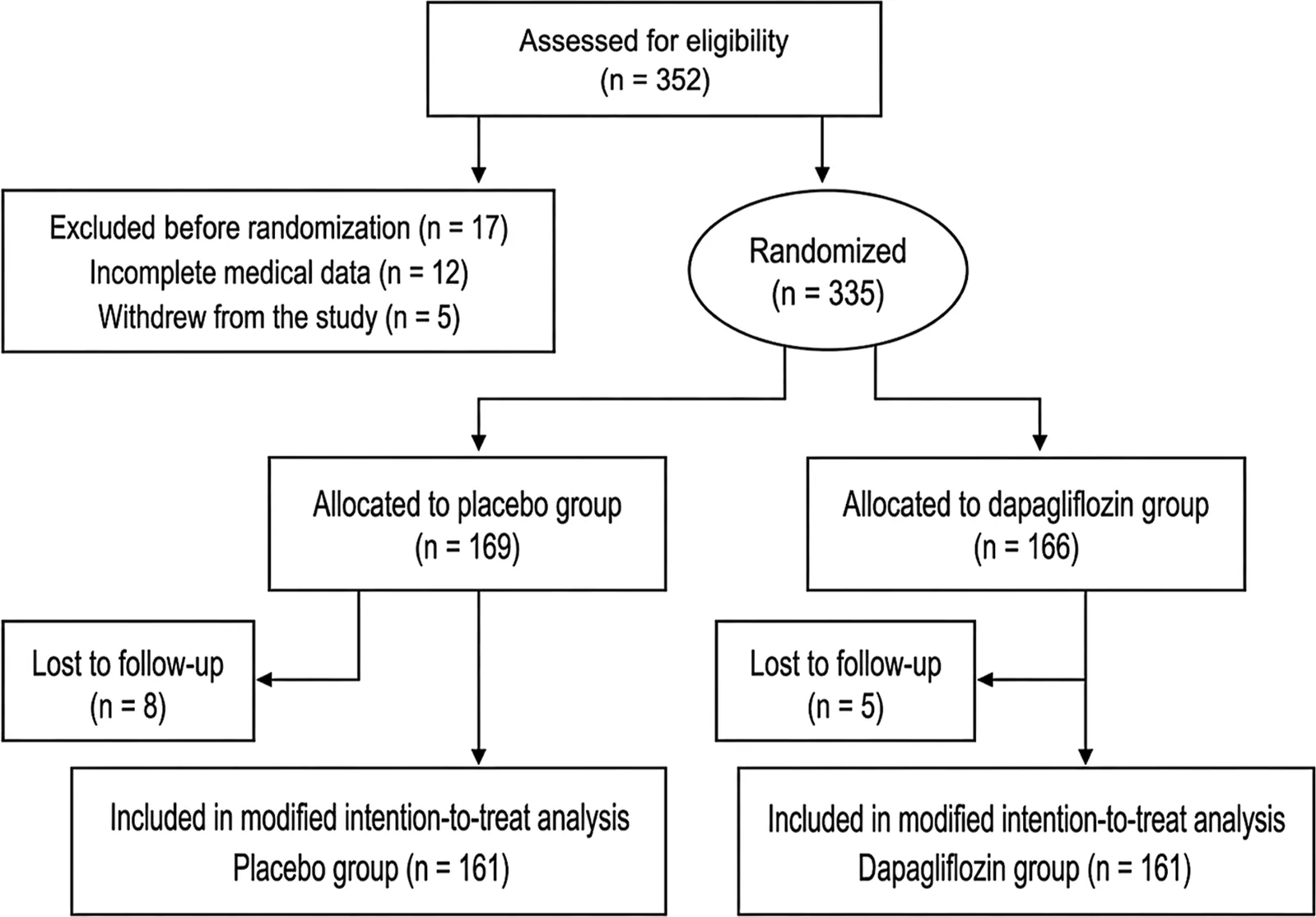
Study flowchart. A total of 352 patients were assessed for eligibility, 335 were randomized, and 322 patients with complete baseline and post-CCTA creatinine assessment were included in the modified intention-to-treat analysis, with 161 patients in each treatment group. CCTA, coronary computed tomographic angiography.

### Inclusion and Exclusion Criteria

Patients were eligible if they were aged 18 years or older, had clinically diagnosed type 2 diabetes mellitus, were scheduled to undergo CCTA for evaluation of chest pain, angina, or suspected coronary artery disease, had stable vital signs, and were able to provide informed consent. Exclusion criteria included severe heart failure defined as New York Heart Association class III or higher, end-stage kidney disease, ongoing kidney replacement therapy, prior or current SGLT2 inhibitor use before enrollment, known allergy to iodinated contrast media.

Patients receiving dialysis or other kidney replacement therapy and those with end-stage kidney disease were excluded, whereas reduced baseline eGFR alone did not constitute an exclusion criterion. Baseline and post-CCTA eGFR values were calculated from the corresponding Scr values [16] and categorized as ≥90, 60–89, 45–59, 30–44, and <30 mL/min/1.73 m^2^ for renal risk stratification. Post-CCTA creatinine changes were additionally classified according to KDIGO creatinine-based AKI stages for severity description [17]. The requirement for new dialysis or kidney replacement therapy during follow-up was verified from electronic medical records.

### Randomization, Blinding, Intervention, and Renal Preventive Measures

Eligible participants were randomized in a 1:1 ratio using computer-generated permuted block randomization. The allocation sequence was generated by an independent investigator not involved in enrollment, treatment, outcome assessment, or data analysis. Sequentially numbered, opaque, sealed envelopes containing group assignments were prepared before enrollment and opened only after eligibility confirmation. Participants, investigators, outcome assessors, and data analysts remained blinded to treatment allocation during study conduct.

Participants received dapagliflozin 10 mg once daily or matched placebo beginning 3 days before CCTA and continuing until completion of the 72-h post-CCTA follow-up. Baseline Scr and eGFR were measured before randomization and before the first dose of study medication, thereby avoiding bias from an early SGLT2 inhibitor-related eGFR dip.

According to institutional practice, prophylactic hydration was routinely provided to patients with baseline eGFR <60 mL/min/1.73 m^2^ or clinically recognized chronic kidney disease, unless contraindicated by overt volume overload, decompensated heart failure, or unstable hemodynamics. In patients with baseline eGFR ≥60 mL/min/1.73 m^2^, prophylactic hydration was not routinely required and was administered only when clinically indicated at the discretion of the treating physician. For patients receiving hydration prophylaxis, the regimen consisted of approximately 500 mL of 0.9% saline before CCTA and 500 mL after CCTA, with adjustment according to cardiac function, baseline renal function, and clinical volume status.

Potentially nephrotoxic or renal hemodynamically active medications were managed using a unified protocol. Metformin, NSAIDs, ACEI/angiotensin receptor blocker, and diuretics were temporarily withheld around the CCTA procedure and recorded in the case report form. Metformin was restarted only after post-procedural renal function was confirmed to be stable. Contrast volume was minimized according to patient body size, scan protocol, heart rate control, and image quality requirements.

### Sample Size Estimation

The sample size was estimated for the primary endpoint of CI-AKI incidence using a two-group comparison of independent proportions. Based on assumptions derived from prior studies and preliminary observations, with a two-sided alpha level of 0.05, 80% power, and a 1:1 allocation ratio, 146 participants were required in each group. After allowing for approximately 10% attrition, the target sample size was increased to 161 participants per group.

### CCTA Acquisition Protocol

All participants underwent CCTA according to the institutional protocol. Scan parameters included a tube voltage of 100–120 kV, tube current of 100–300 mA s, collimation of 128 × 0.625 mm, gantry rotation time of 0.27 s, and a 512 × 512 matrix. For contrast enhancement, a nonionic low-osmolar iodinated contrast agent, ioversol injection (Jiangsu Hengrui Medicine Co., Ltd., Lianyungang, China), was injected at 4–5 mL/s followed by a 50-mL saline flush at the same flow rate. Bolus tracking was performed in the ascending aorta, and scanning was automatically triggered when attenuation reached 150 Hounsfield units after a 6-s delay.

### Clinical Data Collection and Endpoint Definition

Baseline demographic, clinical, laboratory, and medication data were collected using standardized case report forms. Scr was measured at baseline before randomization and before the first dose of study medication, and again at 48 and 72 h after CCTA. CI-AKI was defined according to the prespecified creatinine-based criterion used in the study dataset: an increase in Scr of ≥44.2 μmol/L or ≥25% from baseline within 72 h after contrast exposure. Baseline renal function was defined using the Scr value obtained before initiation of the study medication.

### Statistical Analysis

Continuous variables are presented as mean ± standard deviation or median with interquartile range according to distribution, and categorical variables are presented as *n* (%). Between-group comparisons used the independent sample t test or Mann-Whitney U test for continuous variables and the chi-square test or Fisher’s exact test for categorical variables, as appropriate.

The primary comparison was the incidence of CI-AKI between treatment groups in the modified intention-to-treat population. Serial renal indices and exploratory biomarker variables were summarized at baseline, 48 h, and 72 h after CCTA when available. Between-group differences at prespecified time points and changes from baseline were assessed using appropriate parametric or nonparametric tests according to distribution.

Given the limited number of CI-AKI events, multivariable logistic regression was intentionally parsimonious. Treatment assignment was forced into the model, and additional covariates were limited to clinically prespecified renal and cardiovascular vulnerability markers rather than being selected solely by univariable statistical significance. Variance inflation factors were examined to assess collinearity.

## Results

### Study Population and Baseline Characteristics

Among 352 screened patients, 335 were randomized and 322 patients were included in the modified intention-to-treat analysis, with 161 patients in the dapagliflozin group and 161 in the placebo group. The analysis dataset included complete baseline and post-CCTA creatinine measurements for primary endpoint assessment. Baseline age, sex, hypertension prevalence, Scr, eGFR, and baseline eGFR category distributions were generally comparable between groups, as shown in Table 1. Left ventricular ejection fraction was presented as median (interquartile range) because of its distribution and low-end values, and it was not significantly different between groups (61.0 [55.0–66.0] vs. 59.0 [51.0–67.0]%; *p* = 0.091). Baseline blood urea nitrogen (BUN) and neutrophil gelatinase-associated lipocalin (NGAL) levels were also comparable between groups, as shown in Table 2. These baseline characteristics were considered when interpreting the treatment association.

**Table 1.** Baseline characteristics of the modified intention-to-treat population

Characteristic	Placebo group (*n* = 161)	Dapagliflozin group (*n* = 161)	*p* value
Male sex, *n* (%)	109 (67.7)	103 (64.0)	0.481
Age, years	64.11±10.77	65.93±10.97	0.184
Baseline Scr, μmol/L	86.81±30.78	84.36±28.57	0.376
Baseline eGFR, mL/min/1.73 m^2^	82.30±22.88	83.37±22.93	0.807
Baseline eGFR category, *n* (%)	​	​	0.788
≥90 mL/min/1.73 m^2^	60 (37.3)	64 (39.8)	​
60–89 mL/min/1.73 m^2^	73 (45.3)	72 (44.7)	​
45–59 mL/min/1.73 m^2^	20 (12.4)	17 (10.6)	​
30–44 mL/min/1.73 m^2^	5 (3.1)	7 (4.3)	​
<30 mL/min/1.73 m^2^	3 (1.9)	1 (0.6)	​
Baseline eGFR <60 mL/min/1.73 m^2^, *n* (%)	28 (17.4)	25 (15.5)	0.652
Baseline eGFR <45 mL/min/1.73 m^2^, *n* (%)	8 (5.0)	8 (5.0)	1.000
Baseline eGFR <30 mL/min/1.73 m^2^, *n* (%)	3 (1.9)	1 (0.6)	0.623
History of hypertension, *n* (%)	87 (54.0)	88 (54.7)	0.911
Atrial fibrillation, *n* (%)	15 (9.3)	24 (14.9)	0.124
Fatty liver, *n* (%)	35 (21.7)	31 (19.3)	0.581
Total cholesterol, mmol/L	4.22±1.09	4.40±1.52	0.544
Triglycerides, mmol/L	1.38 (0.97–2.15)	1.53 (1.12–2.24)	0.067
LDL-C, mmol/L	2.25±0.77	2.37±0.95	0.563
Lipoprotein(a), mg/L	45.80 (7.87–147.40)	70.75 (11.91–186.75)	0.119
Myoglobin, μg/L	37.10 (28.93–49.00)	42.00 (28.00–57.18)	0.207
D-dimer, mg/L	0.35 (0.19–0.73)	0.35 (0.23–0.77)	0.152
LVEF, %	61.0 (55.0–66.0)	59.0 (51.0–67.0)	0.091
ACEI/ARB use, *n* (%)	82 (50.9)	98 (60.9)	0.073
CCB use, *n* (%)	64 (39.8)	55 (34.2)	0.299
Beta-blocker use, *n* (%)	71 (44.1)	85 (52.8)	0.118
Metformin use, *n* (%)	6 (3.7)	14 (8.7)	0.065
Statin use, *n* (%)	112 (69.6)	118 (73.3)	0.459
Aspirin use, *n* (%)	90 (55.9)	94 (58.4)	0.652
Clopidogrel use, *n* (%)	52 (32.3)	53 (32.9)	0.905
Acarbose use, *n* (%)	30 (18.6)	37 (23.0)	0.337

Data are presented as mean ± standard deviation, median (interquartile range), or *n* (%), unless otherwise specified. Baseline Scr was measured before randomization and before the first dose of study medication, and baseline eGFR was calculated from the corresponding Scr value. Between-group comparisons used the independent sample *t* test or Mann-Whitney U test for continuous variables according to distribution, and the chi-square or Fisher’s exact test for categorical variables, as appropriate. CCTA, coronary computed tomography angiography; Scr, serum creatinine; eGFR, estimated glomerular filtration rate; LVEF, left ventricular ejection fraction; ACEI, angiotensin-converting enzyme inhibitor; ARB, angiotensin receptor blocker; CCB, calcium channel blocker.

**Table 2.** Renal outcomes and exploratory biomarker profile before and after CCTA

Variable	Placebo group (*n* = 161)	Dapagliflozin group (*n* = 161)	*p* value
Baseline Scr before study medication, μmol/L	86.81±30.78	84.36±28.57	0.376
Scr at 48 h after CCTA, μmol/L	92.05±39.00	83.82±30.09	0.034
Scr at 72 h after CCTA, μmol/L	94.81±48.57	82.86±31.42	0.011
Absolute Scr change at 72 h, μmol/L	8.00±35.51	−1.50±11.21	<0.001
Relative Scr change at 72 h, %	8.62±34.92	−2.22±11.68	<0.001
Baseline eGFR before study medication, mL/min/1.73 m^2^	82.30±22.88	83.37±22.93	0.807
eGFR at 48 h after CCTA, mL/min/1.73 m^2^	79.82±25.75	84.96±24.58	0.099
eGFR at 72 h after CCTA, mL/min/1.73 m^2^	79.74±27.42	87.03±26.35	0.027
Absolute eGFR change at 72 h, mL/min/1.73 m^2^	−2.56±13.93	3.66±7.70	<0.001
Baseline eGFR <60 mL/min/1.73 m^2^, *n* (%)	28 (17.4)	25 (15.5)	0.652
eGFR <60 at 72 h after CCTA, *n* (%)	41 (25.5)	24 (14.9)	0.018
Baseline eGFR <45 mL/min/1.73 m^2^, *n* (%)	8 (5.0)	8 (5.0)	1.000
eGFR <45 at 72 h after CCTA, *n* (%)	17 (10.6)	12 (7.5)	0.330
Baseline eGFR <30 mL/min/1.73 m^2^, *n* (%)	3 (1.9)	1 (0.6)	0.623
eGFR <30 at 72 h after CCTA, *n* (%)	8 (5.0)	3 (1.9)	0.125
Baseline BUN, mmol/L	6.06±1.20	6.02±1.23	0.861
BUN at 72 h after CCTA, mmol/L	7.56±0.81	6.23±0.75	<0.001
Absolute BUN change, mmol/L	1.51±1.31	0.21±1.43	<0.001
Baseline NGAL, ng/mL	83.97±18.67	84.91±18.75	0.805
NGAL at 48 h after CCTA, ng/mL	96.40±21.25	95.35±23.26	0.626
NGAL at 72 h after CCTA, ng/mL	124.20±25.78	112.15±24.56	<0.001
Absolute NGAL change at 48 h, ng/mL	12.43±27.34	10.44±31.06	0.551
Absolute NGAL change at 72 h, ng/mL	40.23±30.89	27.24±31.89	<0.001
Study-defined CI-AKI, *n* (%)	23 (14.3)	6 (3.7)	0.001
New dialysis or kidney replacement therapy within 72 h, *n* (%)	0 (0.0)	0 (0.0)	N/A
KDIGO creatinine stage after CCTA, *n* (%)	​	​	0.064
No KDIGO AKI	147 (91.3)	157 (97.5)	​
Stage 1	9 (5.6)	4 (2.5)	​
Stage 2	2 (1.2)	0 (0.0)	​
Stage 3	3 (1.9)	0 (0.0)	​

Data are presented as mean ± standard deviation or *n* (%), unless otherwise specified. Baseline Scr was measured before randomization and before the first dose of study medication; baseline eGFR and baseline eGFR categories were calculated from the corresponding Scr value. Scr values at 48 and 72 h after CCTA were obtained from the clinical dataset, and post-CCTA eGFR values were calculated from the corresponding Scr values. CI-AKI was defined according to individual patient-level creatinine changes within 72 h after contrast exposure. For the primary endpoint, the unadjusted OR for dapagliflozin vs. placebo was 0.23 (95% CI, 0.09–0.59), and the *p* value of 0.001 was obtained using Fisher’s exact test. KDIGO staging was recalculated from the current dataset. NGAL values are shown in ng/mL; the values are numerically equivalent to μg/L. CCTA, coronary computed tomography angiography; Scr, serum creatinine; eGFR, estimated glomerular filtration rate; BUN, blood urea nitrogen; NGAL, neutrophil gelatinase-associated lipocalin; CI-AKI, contrast-induced acute kidney injury.

Based on baseline eGFR categories, 124 patients had eGFR ≥90 mL/min/1.73 m^2^, 145 had eGFR 60–89 mL/min/1.73 m^2^, 37 had eGFR 45–59 mL/min/1.73 m^2^, 12 had eGFR 30–44 mL/min/1.73 m^2^, and 4 had eGFR <30 mL/min/1.73 m^2^. Overall, 53 of 322 patients (16.5%) had baseline eGFR <60 mL/min/1.73 m^2^, 16 patients (5.0%) had eGFR <45 mL/min/1.73 m^2^, and 4 patients (1.2%) had eGFR <30 mL/min/1.73 m^2^.

### Incidence and Severity of CI-AKI

CI-AKI occurred in 29 of 322 patients (9.0%). The incidence was significantly lower in the dapagliflozin group than in the placebo group (6/161 [3.7%] vs. 23/161 [14.3%]; odds ratio [OR], 0.23; 95% confidence interval, 0.09–0.59; *p* = 0.001 by Fisher’s exact test). No patient required new dialysis or kidney replacement therapy during the 72-h post-CCTA follow-up.

According to KDIGO creatinine-based severity classification after CCTA, most patients did not meet KDIGO AKI criteria. No KDIGO AKI was observed in 147 patients (91.3%) in the placebo group and 157 patients (97.5%) in the dapagliflozin group. KDIGO stage 1 occurred in 9 patients (5.6%) and 4 patients (2.5%), stage 2 in 2 patients (1.2%) and 0 patients, and stage 3 in 3 patients (1.9%) and 0 patients, respectively (*p* = 0.064). Some patients met the prespecified CI-AKI definition based on a ≥25% creatinine increase but did not meet KDIGO stage 1 criteria. Thus, KDIGO staging was used to describe severity, whereas the primary endpoint was defined according to the prespecified study criterion. The renal outcomes and KDIGO creatinine stage distribution are summarized in Table 2.

### Renal Function Trajectory after CCTA

Group-level Scr and eGFR trajectories were directionally consistent after CCTA. Baseline Scr, measured before randomization and the first dose of study medication, was comparable between the placebo and dapagliflozin groups (86.81 ± 30.78 vs. 84.36 ± 28.57 μmol/L; *p* = 0.376). At 72 h after CCTA, Scr was higher in the placebo group than in the dapagliflozin group (94.81 ± 48.57 vs. 82.86 ± 31.42 μmol/L; *p* = 0.011). The absolute Scr change at 72 h was 8.00 ± 35.51 μmol/L in the placebo group and −1.50 ± 11.21 μmol/L in the dapagliflozin group (*p* < 0.001). Baseline eGFR, calculated from the corresponding baseline Scr value, was also comparable between groups (82.30 ± 22.88 vs. 83.37 ± 22.93 mL/min/1.73 m^2^; *p* = 0.807). At 72 h, eGFR was lower in the placebo group than in the dapagliflozin group (79.74 ± 27.42 vs. 87.03 ± 26.35 mL/min/1.73 m^2^; *p* = 0.027), with an absolute eGFR change of −2.56 ± 13.93 vs. 3.66 ± 7.70 mL/min/1.73 m^2^ (*p* < 0.001).

The primary endpoint was defined by individual patient-level creatinine changes rather than by group-level mean creatinine or eGFR changes. Therefore, patients could meet the CI-AKI endpoint despite modest average changes at the cohort level. Baseline BUN and NGAL were comparable between groups. At 72 h after CCTA, BUN was higher in the placebo group than in the dapagliflozin group (7.56 ± 0.81 vs. 6.23 ± 0.75 mmol/L; *p* < 0.001), and NGAL was also higher in the placebo group (124.20 ± 25.78 vs. 112.15 ± 24.56 ng/mL; *p* < 0.001) as shown in Table 2.

### Factors Associated with CI-AKI

Logistic regression was performed to explore clinical factors associated with CI-AKI. Because only 29 events occurred, the multivariable model was restricted to a small prespecified set of covariates to reduce overfitting. In the parsimonious model, dapagliflozin use remained associated with a lower risk of CI-AKI (adjusted OR, 0.17; 95% CI, 0.07–0.46; *p* < 0.001), whereas older age was associated with a higher risk (adjusted OR per year, 1.09; 95% CI, 1.04–1.14; *p* < 0.001). Baseline eGFR <60 mL/min/1.73 m^2^ was not statistically associated with CI-AKI (adjusted OR, 0.33; 95% CI, 0.07–1.48; *p* = 0.147). Variance inflation factors did not suggest meaningful collinearity among the included covariates. The univariable and parsimonious multivariable logistic regression results are presented in Table 3.

**Table 3.** Exploratory univariable and parsimonious multivariable logistic regression analyses for study-defined CI-AKI

Variable	Univariable OR	95% CI	*p* value	Multivariable OR	95% CI	*p* value
Dapagliflozin use	0.23	0.09–0.59	0.002	0.17	0.07–0.46	<0.001
Age, per year	1.08	1.03–1.13	0.001	1.09	1.04–1.14	<0.001
Male sex	0.61	0.28–1.32	0.208	—	—	—
Baseline eGFR <60 mL/min/1.73 m^2^	0.35	0.08–1.53	0.163	0.33	0.07–1.48	0.147
Baseline Scr, per 10 μmol/L	0.91	0.77–1.07	0.237	—	—	—
History of hypertension	1.42	0.65–3.11	0.383	—	—	—
Atrial fibrillation	1.59	0.57–4.44	0.378	—	—	—
ACEI/ARB use	0.97	0.45–2.09	0.934	—	—	—
CCB use	1.05	0.48–2.30	0.909	—	—	—
Beta-blocker use	0.62	0.28–1.37	0.238	—	—	—
Metformin use	1.87	0.51–6.82	0.341	—	—	—
Statin use	0.63	0.28–1.38	0.245	—	—	—
Aspirin use	0.68	0.31–1.45	0.314	—	—	—
Clopidogrel use	0.92	0.41–2.10	0.850	—	—	—
Acarbose use	0.78	0.28–2.12	0.621	—	—	—
LVEF, per 5%	0.90	0.76–1.06	0.196	—	—	—

ORs and 95% confidence intervals were estimated using logistic regression in the modified intention-to-treat population (*n* = 322; 29 CI-AKI events). The multivariable model was restricted to dapagliflozin use, age, and baseline eGFR <60 mL/min/1.73 m^2^. The corresponding variance inflation factors were 1.01, 1.01, and 1.00, respectively. The *p* value for the primary unadjusted between-group comparison was obtained using Fisher’s exact test (*p* = 0.001), whereas the *p* value shown for dapagliflozin use in the univariable logistic regression was based on the Wald test (*p* = 0.002). CI-AKI, contrast-induced acute kidney injury; OR, odds ratio; CI, confidence interval; LVEF, left ventricular ejection fraction; ARB, angiotensin receptor blocker.

## Discussion

In this prospective randomized double-blind placebo-controlled study, short-term peri-procedural dapagliflozin was associated with a lower incidence of creatinine-defined CI-AKI in diabetic patients undergoing CCTA. The overall incidence of CI-AKI was 9.0%, with fewer events in the dapagliflozin group than in the placebo group. The dapagliflozin group also showed more favorable early renal function profiles, including lower 72-h Scr, higher 72-h eGFR, and lower exploratory BUN and NGAL levels. These findings suggest that short-term SGLT2 inhibition may provide a clinically relevant renal protective signal in selected diabetic patients undergoing contrast-enhanced cardiac CT.

A key strength and novelty of the present study lies in its clinical setting. Most previous studies evaluating SGLT2 inhibitors and contrast-associated kidney injury have focused on invasive coronary angiography or PCI [14, 18]. In those settings, renal outcomes may be affected by procedural complexity, peri-procedural hemodynamic fluctuation, ischemic burden, emergency presentation, antithrombotic therapy, and multiple concomitant exposures. In contrast, CCTA represents a noninvasive intravenous contrast setting with more standardized contrast administration and substantially less procedure-related hemodynamic stress [19–21]. Contemporary radiology and nephrology perspectives also emphasize that the nephrotoxic risk of intravenous iodinated contrast media has likely been overstated in some settings, while individualized risk assessment remains important in patients with impaired kidney function or additional susceptibility factors [22]. Therefore, CCTA provides a distinct and clinically relevant model for evaluating the interaction among diabetic susceptibility, iodinated contrast exposure, and pharmacological renal protection. By focusing on this population, the present study extends the investigation of peri-procedural SGLT2 inhibition beyond invasive coronary procedures and addresses a preventive strategy that remains insufficiently studied in contrast-enhanced cardiac CT.

The present findings are also consistent with the concept that renal risk after intravenous contrast exposure is heterogeneous rather than uniformly low. In contrast-enhanced CT populations, Lee et al. [23] showed that diabetes status and kidney function were important determinants of AKI risk. In diabetic patients undergoing CCTA, Isobe et al. [24] reported that pre-procedural microalbuminuria was associated with renal functional changes after coronary CT, suggesting that eGFR alone may not fully capture diabetic renal microvascular vulnerability. More broadly, diabetes has been recognized as a distinct risk factor for AKI through pathways involving renal microvascular dysfunction, endothelial injury, oxidative stress, tubular metabolic stress, and impaired autoregulatory responses [25]. These observations provide important context for the present cohort, in which CI-AKI risk appeared to reflect broader cardiometabolic and renal susceptibility rather than reduced eGFR alone.

Evidence from invasive coronary procedures provides a relevant but not directly equivalent comparator. A systematic review and meta-analysis suggested that SGLT2 inhibitor use was associated with a lower risk of contrast-induced nephropathy in patients with diabetes undergoing coronary angiography or PCI [14]. Similarly, a randomized trial of short-term empagliflozin in PCI patients and observational studies of SGLT2 inhibitor or dapagliflozin use in coronary intervention settings reported lower CI-AKI risk or favorable cardiorenal signals [18, 26, 27]. Conversely, Yang et al. [28] reported that short-term dapagliflozin administration before PCI was associated with a higher incidence of CI-AKI in patients with type 2 diabetes and renal insufficiency. These apparently divergent findings highlight the importance of procedural context, baseline renal reserve, hydration status, treatment timing, and patient selection. Compared with these prior studies, the present trial specifically evaluates a short peri-procedural dapagliflozin regimen in a noninvasive CCTA population, thereby providing complementary evidence for an intravenous contrast-enhanced cardiac CT setting.

The relatively high CI-AKI incidence in the present study warrants interpretation in light of the enrolled population and endpoint definition. An overall incidence of 9.0% is higher than would be expected in an unselected low-risk outpatient CT population, but it is clinically plausible in an enriched diabetic cohort referred for CCTA. Importantly, this event rate should not be attributed solely to reduced baseline eGFR. Although 53 of 322 patients had baseline eGFR <60 mL/min/1.73 m^2^, the observed CI-AKI incidence may not be explained by reduced baseline eGFR alone. Instead, the observed incidence likely reflects several risk dimensions, including diabetes-related renal microvascular vulnerability, possible unmeasured albuminuria or proteinuria, broader cardiorenal-metabolic risk, repeated creatinine ascertainment, and use of the prespecified ≥25% creatinine increase criterion. Published estimates of post-contrast AKI after intravenous contrast CT vary according to baseline risk, diabetes status, inpatient status, endpoint definition, and timing of creatinine testing [29]. Therefore, the present event rate should be interpreted as applying to diabetic patients undergoing CCTA with appreciable metabolic and renal vulnerability rather than to all patients undergoing diagnostic CT. The absence of new dialysis or kidney replacement therapy during follow-up further suggests that most events represented early creatinine-defined renal function changes rather than severe renal failure.

Another important aspect of this study is that dapagliflozin was associated with lower CI-AKI incidence despite a short peri-procedural treatment window. SGLT2 inhibitors are well established for long-term cardiorenal protection in patients with diabetes, heart failure, and chronic kidney disease [30, 31]. The present findings suggest that selected renal protective effects may also emerge over a shorter time frame around contrast exposure. Because dapagliflozin was initiated before CCTA and continued through the 72-h post-contrast period, the observed association is more likely to reflect early functional, hemodynamic, metabolic, or adaptive responses rather than long-term structural renal remodeling. This interpretation is consistent with broader clinical evidence suggesting that SGLT2 inhibitors may reduce AKI risk across patients with type 2 diabetes, cardiovascular disease, heart failure, or chronic kidney disease, although the magnitude and direction of benefit may vary according to baseline renal function and clinical context [13, 14].

Several biological mechanisms may plausibly explain the short-term renal signal observed in the present study. Contrast-associated renal injury is a multifactorial process involving renal parenchymal hypoxia, medullary microcirculatory disturbance, direct tubular toxicity, oxidative stress, endothelial dysfunction, and inflammatory activation, particularly in patients with diabetes [32]. The renal outer medulla is especially vulnerable because the medullary thick ascending limb has high transport-related oxygen demand but relatively low baseline oxygen tension [33]. Against this pathophysiological background, SGLT2 inhibition may confer renoprotective effects through several complementary pathways. By reducing proximal tubular sodium-glucose reabsorption, SGLT2 inhibitors increase sodium delivery to the macula densa, restore tubuloglomerular feedback, and lower intraglomerular pressure [34]. Reduced proximal tubular solute transport may also decrease Na+/K+-ATPase-dependent energy demand and oxygen consumption, thereby improving the balance between oxygen supply and demand in metabolically vulnerable tubular regions. In addition, SGLT2 inhibitors have been associated with attenuation of oxidative stress, inflammatory signaling, endothelial dysfunction, and mitochondrial stress, all of which are relevant to contrast-associated tubular injury [35].

Nevertheless, the renal oxygenation effect of SGLT2 inhibition during acute contrast exposure may be context-dependent. Although reduced proximal tubular workload may lower oxygen consumption, increased distal sodium delivery could theoretically shift transport demand toward downstream nephron segments under certain acute conditions [15, 36]. Therefore, the net effect of peri-procedural SGLT2 inhibition may depend on treatment timing, hydration status, contrast burden, baseline renal reserve, and individual susceptibility. Experimental evidence further supports a potential mechanistic link between dapagliflozin and modulation of hypoxia- and inflammation-related pathways. Huang et al. [37] reported that dapagliflozin attenuated CI-AKI in an experimental model through regulation of the HIF-1α/HE4/NF-κB pathway. Taken together, these findings provide biological plausibility for the observed clinical association, but the present randomized study should be interpreted as showing a short-term clinical treatment signal rather than providing definitive mechanistic proof.

Beyond these mechanistic considerations, the regression and biomarker findings further contextualize the observed treatment signal. Previous PCI studies have identified clinical vulnerability, pre-procedural biomarkers, and post-procedural AKI itself as prognostically relevant in contrast-exposed coronary populations [38–40]. In the present parsimonious multivariable model, dapagliflozin use remained associated with lower CI-AKI risk, whereas older age was associated with higher risk. Baseline eGFR <60 mL/min/1.73 m^2^ was not statistically associated with CI-AKI in this cohort; however, patients with impaired baseline renal function preferentially received prophylactic hydration according to institutional practice. This finding should therefore not be interpreted as evidence that reduced eGFR was protective or clinically unimportant. Because of the limited number of events, the multivariable analysis should be interpreted as supportive rather than as a definitive risk-prediction model. Similarly, lower 72-h BUN and NGAL levels in the dapagliflozin group provide exploratory biological context, but NGAL was not measured at very early post-contrast time points such as 2–6 h after CCTA [41]. Therefore, the principal inference remains anchored to the prespecified creatinine-based CI-AKI endpoint [3, 17].

From a clinical perspective, these findings suggest that short-term peri-procedural dapagliflozin may represent a novel adjunctive renal risk reduction strategy for selected diabetic patients undergoing CCTA. This is clinically relevant because renal preventive strategies in noninvasive intravenous contrast-enhanced cardiac CT remain less well defined than in invasive coronary procedures. Nevertheless, dapagliflozin should be considered complementary to, rather than a replacement for, established preventive measures, including risk identification, optimization of volume status, hydration when appropriate, contrast minimization, avoidance of unnecessary nephrotoxic exposures, and standardized metformin management [42, 43]. The clinical need for adjunctive approaches is further supported by trials showing limitations of traditional prophylactic strategies. In the PRESERVE trial, intravenous sodium bicarbonate and oral acetylcysteine did not improve major kidney outcomes compared with isotonic saline or placebo in high-risk patients undergoing angiography [44]. Similarly, the AMACING trial challenged routine prophylactic hydration in selected patients at risk of contrast-induced nephropathy [45]. These findings reinforce the importance of individualized risk assessment and the search for adjunctive strategies with a more specific pathophysiological rationale.

Several limitations should be acknowledged. First, the principal limitation of this study is the very small fraction of patients with baseline eGFR <30 mL/min/1.73 m^2^. Only 4 patients (1.2%) had baseline eGFR <30 mL/min/1.73 m^2^, which limits the applicability of our findings to patients with advanced chronic kidney disease. This is an important consideration because patients with severely reduced renal function represent the subgroup in whom the risk of CI-AKI after contrast-enhanced CT appears most clinically relevant and has been more clearly demonstrated, including in the study by Gorelik et al. [46]. Therefore, the present findings should not be extrapolated to patients with advanced chronic kidney disease or eGFR <30 mL/min/1.73 m^2^. Second, this was a single-center study with a moderate sample size and a limited number of CI-AKI events, which may restrict generalizability and limit the complexity of multivariable modeling. Third, the primary analysis included patients with available post-CCTA creatinine measurements, and missing endpoint ascertainment may have introduced selection bias. Fourth, follow-up was limited to 72 h after contrast exposure; therefore, longer term renal and cardiovascular outcomes could not be evaluated. Fifth, Scr is a delayed functional marker, and the exploratory BUN and NGAL analyses cannot definitively distinguish transient hemodynamic changes from structural tubular injury, particularly because NGAL was not measured at very early post-contrast time points. Sixth, urinary albumin-to-creatinine ratio, renal medullary oxygenation, renal microcirculation, oxidative stress markers, and HIF-related biomarkers were not systematically assessed, limiting mechanistic interpretation. Finally, these findings should not be extrapolated beyond short-term peri-procedural dapagliflozin use in diabetic patients undergoing CCTA. Larger multicenter studies are needed to confirm these findings, define the optimal timing and safety profile, and identify the patients most likely to benefit.

## Conclusion

Short-term peri-procedural dapagliflozin was associated with a lower incidence of creatinine-defined CI-AKI and more favorable early renal function profiles in diabetic patients undergoing CCTA. By focusing on a noninvasive intravenous contrast-enhanced cardiac CT population, this study extends the potential application of SGLT2 inhibition beyond invasive coronary procedures and suggests a novel adjunctive approach for renal risk reduction in selected diabetic patients. Larger multicenter studies are warranted to validate these findings, define the optimal timing and safety profile, and identify the patients most likely to benefit.

## Acknowledgments

The authors thank all staff members who contributed to study implementation and patient evaluation.

## Statement of Ethics

The study protocol was approved by the Institutional Review Board of the Second Affiliated Hospital of Nanchang University (Approval No. N-202007038). All procedures complied with the Declaration of Helsinki and its later amendments. Written informed consent was obtained from all participants before enrollment.

## Conflict of Interest Statement

The authors declare no conflict of interest.

## Funding Sources

This work was supported by the National Natural Science Foundation of China [82060075], the Jiangxi Provincial Natural Science Foundation [20212BAB216057], the Second Affiliated Hospital of Nanchang University Funding Program [2023efyA01], the Jiangxi Provincial Health Commission Science and Technology Project [202410605], the Science and Technology Plan of Jiangxi Provincial Administration of Traditional Chinese Medicine [2023A0380], and the Jiangxi Administration of Traditional Chinese Medicine Science and Technology Project [2022B1053]. These funding sources provided financial support for the implementation of the study, including research-related expenses, data collection and management, statistical analysis, and manuscript preparation. The funding bodies had no direct role in the study design, patient recruitment, study execution, data analysis, data interpretation, manuscript conception, manuscript planning, manuscript writing, or the decision to submit the manuscript for publication. The authors were solely responsible for the study conduct, interpretation of the findings, and the final decision to publish.

## Author Contributions

Tian Zheng and Lifeng Zhang contributed to the study conception and design, data collection, data analysis, and drafting of the manuscript. Jianxin Hu, Xiaoshu Cheng, Yuxuan Zhong, and Xiao Huang contributed to patient recruitment, clinical data collection, and follow-up assessments. Lianggeng Gong contributed to data interpretation, statistical analysis, and critical revision of the manuscript. Biming Zhan supervised the study, contributed to the study design, interpreted the findings, and critically revised the manuscript for important intellectual content. All authors read and approved the final version of the manuscript.

## Data Availability

The data that support the findings of this study are not publicly available because of patient privacy and institutional data protection requirements but are available from the corresponding author upon reasonable request and with appropriate ethical approval.
